# Combining robust level extraction and unsupervised adaptive classification for high-accuracy fNIRS-BCI: An evidence on single-trial differentiation between mentally arithmetic- and singing-tasks

**DOI:** 10.3389/fnins.2022.938518

**Published:** 2022-10-10

**Authors:** Yao Zhang, Dongyuan Liu, Pengrui Zhang, Tieni Li, Zhiyong Li, Feng Gao

**Affiliations:** ^1^College of Precision Instrument and Optoelectronics Engineering, Tianjin University, Tianjin, China; ^2^Tianjin Key Laboratory of Biomedical Detecting Techniques and Instruments, Tianjin University, Tianjin, China

**Keywords:** activation level, adaptive Gaussian mixture model, brain-computer interface, classification accuracy, fNIRS, feature extraction, general linear model, Kalman filtering

## Abstract

Functional near-infrared spectroscopy (fNIRS) is a safe and non-invasive optical imaging technique that is being increasingly used in brain-computer interfaces (BCIs) to recognize mental tasks. Unlike electroencephalography (EEG) which directly measures neural activation, fNIRS signals reflect neurovascular-coupling inducing hemodynamic response that can be slow in time and varying in the pattern. The established classifiers extend the EEG-ones by mostly employing the feature based supervised models such as the support vector machine (SVM) and linear discriminant analysis (LDA), and fail to timely characterize the level-sensitive hemodynamic pattern. A dedicated classifier is desired for intentional activity recognition of fNIRS-BCI, including the adaptive acquisition of response relevant features and accurate discrimination of implied ideas. To this end, we herein propose a specifically-designed joint adaptive classification method that combines a Kalman filtering (KF) for robust level extraction and an adaptive Gaussian mixture model (a-GMM) for enhanced pattern recognition. The simulative investigations and paradigm experiments have shown that the proposed KF/a-GMM classification method can effectively track the random variations of task-evoked brain activation patterns, and improve the accuracy of single-trial classification task of mental arithmetic vs. mental singing, as compared to the conventional methods, e.g., those that employ combinations of the band-pass filtering (BPF) based feature extractors (mean, slope, and variance, etc.) and the classical recognizers (GMM, SVM, and LDA). The proposed approach paves a promising way for developing the real-time fNIRS-BCI technique.

## Introduction

Functional near-infrared spectroscopy (fNIRS) is a non-invasive, safe, more portable, low-motion artifact, and low-cost optical neural imaging technique that measures the cerebral hemodynamic changes associated with functional brain activity in multiple channels while people performing a wide range of mental tasks (Ferrari and Quaresima, 2012; Alzahab et al., 2021). The regional cerebral blood flow variation is caused by the concentration variation of oxygenated hemoglobin (HbO) and deoxygenated hemoglobin (HbR), which are primary absorbing chromophores in the capillaries of the brain (Boas et al., 2004). These days, many brain imaging modalities have been investigated for use in brain-computer interface (BCI). Nevertheless, fNIRS has received an enormous amount of attention due to its superior environmental robustness to EEG (Hong et al., 2020), and is silent and more tolerant to subtle movement artifacts than functional magnetic resonance imaging (fMRI) (Glover, 1999). Another notable advantage of fNIRS-BCI is its suitability for repeated measurements within short intervals and long-term continuous measurements for future clinical use (Li et al., 2017).

To measure the fNIRS signal, the optodes require direct contact with the scalp, as hair (especially dark hair) leads to attenuation of the fNIRS signal (Herff et al., 2013). However, hair-covered regions of interest (ROI) such as the motor and visual cortex regions have difficulty meeting this condition, as a long preparation time may be required to remove the hair from under the optodes before the experiment to ensure that there is the minimum amount of hair is under the optode. Nevertheless, the hair-free prefrontal cortex (PFC) region is an ideal ROI for fNIRS measurements because it does not cause attenuation of light intensity and allows for a fast set-up of optode layout. In studies where the ROI is the PFC region, fNIRS signal classification is essential to the development of the fNIRS-BCI system. The classification of fNIRS signals acquired in the PFC has applications in many fields, including volitional control such as motor imagery (MI) (Ma et al., 2021), the identification of different emotions (Nguyen et al., 2021), the classification of mental workload levels (Lim et al., 2020), and the discrimination of intentional activity of the brain such as different mental tasks (Power et al., 2012; Chen et al., 2020). Most studies of fNIRS-BCI have focused on MI, affective responses, and mental workload, and less on mental task recognition. In this paper, to investigate the suitability of different mental tasks for BCI control and to improve their discrimination accuracy, we conducted experiments on two mental tasks, namely mental arithmetic (MA) and mental singing (MS). This is because MA and MS are common and robust mental tasks in fNIRS-BCI (Power et al., 2010). Among the studies on the classification of mental tasks, Power et al. (2010) investigated a Hidden Markov Model (HMM) classifier based on light intensity data to classify MA and MS mental tasks. The results of the study showed an average accuracy of 77.2% in 10 able-bodied participants. The results suggest the potential of a two-choice fNIRS-BCI based on mental tasks. In another study, Power et al. (2012) investigated LDA classifiers constructed from feature sets of slope and amplitude to classify the MA vs. MS vs. no-control state of seven able-bodied adults, and the results indicated an overall accuracy of 56.2%. Excluding the ineffective MS task, the accuracy of the three untrained subjects was approaching 70%, which is generally considered effective for binary BCI communication. When users use and practice the fNIRS-BCI system to operate external devices through mental tasks for prolonged periods of time, factors such as learning effects and cognitive fatigue across BCI trials may lead to slow variations in activation patterns over time, which is leading to a reduction in accuracy. The goal of the currently study was to overcome these dilemmas and achieve accurately capturing changes in activation patterns and improving the accuracy of mental tasks. Feature extraction techniques and classification models are essential for improving accuracy. For fNIRS signals, immediate characterization of level-sensitive hemodynamic patterns is critical to improve identification accuracy. Currently, fNIRS-BCI researchers have mostly used various classification models based on statistical features to enhance the classification accuracy of fNIRS signal from ROI.

For using different feature extraction methods to improve accuracy, the most used feature extraction techniques rely on the use of BPF reconstructed hemodynamic response function (HRF) to extract the statistical characteristics of the task-related time-domain fNIRS signals, such as mean, slope, variance, skewness, and kurtosis, and so on (Hwang et al., 2016; Noori et al., 2017; Aydin, 2020). Hwang et al. used the Fisher score method to select the best individual statistical feature for each participant to construct the LDA classifier. The results showed that the average accuracy of “yes” and “no” intentions for the eight healthy participants was ~75% when using the best individual features (Hwang et al., 2016). However, these statistical features cannot well characterize the hemodynamic response in fNIRS-BCI that is fully depicted by the time-varying activation levels. However, the highest accuracy may depend on the participant-specific BPF-statistical features set and the size of the selected time window. One limitation of fNIRS-BCI is that it is time-consuming to process the fNIRS signal to determine the optimal subset of features. Therefore, more suitable adaptive feature extraction techniques are required to overcome the limitations of BPF-statistical features that cannot well characterize the HRF and the time-consuming optimization of a subset of BPF-statistical features. For using different classifiers to improve accuracy, many researchers have tried to apply many machine learning-based classifiers in fNIRS-BCI, such as LDA, SVM, HMM, *k*-nearest neighbor, Gaussian mixture model (GMM), and artificial neural networks (Power et al., 2010; Li et al., 2017; Zhang et al., 2018; Aydin, 2020; Hong et al., 2020), and also tried to apply convolutional neural networks, recurrent neural networks, and other deep learning (DL) algorithms (Trakoolwilaiwan et al., 2018; Asgher et al., 2020; Wickramaratne and Mahmud, 2021), but limited studies are available so far. Yoo et al. developed a long short-term memory network (LSTM) classifier to classify three categories of mental tasks (MA, mental counting, and puzzle solving) (Yoo et al., 2018). The results indicated that the maximum accuracy of the LSTM was 83.30%, which was higher than that of the LDA (37.50%) and SVM (37.96%) classifiers. Although DL methods can automatically extract features and provide improvements in classification performance, a non-negligible problem is that DL usually requires a large amount of data to allow the model to be adequately trained to prevent overfitting. However, it is unrealistic, or even impossible, to obtain substantial-scale labeled fNIRS signals. Therefore, the classifiers currently used in fNIRS-BCI mostly employ the BPF-statistical features based supervised classical models such as SVM and LDA. These EEG-extending methods fail to characterize the HRF and trace changes in activation patterns. For long-term measurements of fNIRS signals, the fNIRS-BCI desires the design of a dedicated adaptive classification method that includes adaptive extraction of the activation level feature and accurate discrimination of the intentional activities, which can capture the changes in the neural activation pattern of users when they use and practice a BCI system.

To this end, we herein propose a novel joint adaptive classification approach called KF/a-GMM that combines the KF method for robust level extraction and the unsupervised a-GMM classification method for accuracy-enhanced pattern recognition. The KF robustly extracts the activation level based on the general linear observational model and the Gaussian-Markov dynamic model, while the a-GMM adaptively classifies the mental tasks through pattern changes-based parameter adjustment. The general linear model (GLM) has been established as a standard method for fMRI data analysis and has also been applied to fNIRS studies using task-based and event-related experimental designs (Schroeter et al., 2004; Abdelnour and Huppert, 2009; Hu et al., 2010). In our model, the KF is used to adaptively estimate the weight coefficients in GLM from all channels of fNIRS data in parallel (Welch and Bishop, 1995; Hu et al., 2010). The HRF-related coefficient estimated at the last time step of a given channel is the extracted activation level feature. The a-GMM has been successfully applied to signal processing in neuroscience and has achieved excellent classification results (Li et al., 2017; Cao et al., 2021). A recent study made by Li et al. showed that an a-GMM classifier could track activation pattern changes without requiring the true labels of the input data (Li et al., 2017). In this paper, we utilize an unsupervised adaptive GMM method, abbreviated as a-GMM, with a transition model that has managed hyper-parameters to adaptively classify different mental tasks. However, the study by Li et al. may be inadequate in simulations, as they only performed single-pattern variation studies and only extracted mean features for pattern recognition (Li et al., 2017). Glover et al. investigated the temporal characteristics of BOLD responses in the sensorimotor and auditory cortex during finger tapping while subjects performed listening to the metronome pacing tones (Glover, 1999). Slow changes and shifts in the size and center of activation regions over time were observed in the dynamic brain activation map of motor and auditory cortical regions, respectively. A similar phenomenon was observed in the dynamic brain activation maps of pairing and transphrasing stimuli in Lin et al.'s study of fNIRS-based Chinese-English simultaneous interpretation (Lin et al., 2018). These changes in neural activation patterns were also studied in simulations by Li et al. (2017). Furthermore, considering that learning effects and cross-trial cognitive fatigue may lead to changes in hemodynamic patterns, it is reasonable to generalize to a possible stochastic form of real-world changes in hemodynamic patterns induced by prolonged mental task stimuli. Therefore, in the present study, more complex randomly varying activation patterns were simulated rather than single-pattern changes. In addition, related *in-vivo* paradigm experiments were performed to validate the classification performance of the proposed method in recognizing different mental tasks.

To demonstrate the efficiency and superiority of the proposed KF/a-GMM approach for high-accuracy classification in mental tasks for fNIRS-BCI, simulation experiments and paradigm experiments are performed to compare mostly employ the BPF-statistical features based classical methods such as GMM, SVM, and LDA. A total of six simulations with randomly varying activation patterns over time are simulated to mimic the possible random variations more realistically in the spatial patterns of neural activation evoked by the two different tasks (i.e., MA vs. MS). These simulations incorporate random walks in the center of the activation region, random variations in the size of the activation region, and random variations in the amplitude of the hemodynamic response. The *in-vivo* paradigm experiments are performed in single-trial classification between the MA vs. MS mental tasks from the prefrontal activity in eight healthy participants.

## Methods

### General linear model

In fNIRS-based studies, changes in the concentrations of HbO and HbR (i.e., Δ[HbO] and Δ[HbR]) can reflect changes in regional cerebral blood flow (rCBF). Since more pronounced amplitude changes of Δ[HbO] can more sensitively reflect the changes of rCBF. Therefore, only Δ[HbO] was considered in subsequent studies. For a given measurement channel, using GLM to analyze the time series of Δ[HbO] signal (Abdelnour and Huppert, 2009). The least-squares method (LSM) is generally used to solve the coefficient **β** in the GLM. The **β** constructed by the LSM method is shown below (Hu et al., 2010):


(1) $$\begin{array}{l} \beta_{LSM}={\left({\text{X}}^{\text{T}}\text{X}\right)}^{-1}{\text{X}}^{\text{T}}\Delta \text{c} \end{array}$$

where Δ **c** ∈ ℝ^*T*×1^ is the *T*-point time series of observed raw Δ[HbO] signal (i.e., unfiltered Δ[HbO] signal), **X** ∈ ℝ^*T*×*L*^ is the design matrix in GLM with five explanatory variables, including HRF, heartbeat signal, Mayer wave signal, respiration signal, and constant (baseline drift). The frequencies of the heartbeat, respiration, and Mayer wave are selected based on the spectral analysis of the raw light intensity, as described in Section Feature extraction. *L* is the total number of explanatory variables (*L* = 5). $\beta_{LSM}\in {\mathbb{R}}^{L\times 1}$ is an unbiased estimate of **β** with the minimum variance according to the Gaussian-Markov theorem (Abdelnour and Huppert, 2009). However, this method requires complete measurement data to calculate **β** but fails the real-time estimation (Wang et al., 2018). In this study, we use KF to recursively estimate the state value **β** at each time step.

### Kalman filtering

The KF method is an adaptive tracking scheme that performs an optimal estimation of the state of a process using a recursively regularized linear inversion routine (Kalman, 1960). In the present study, the KF is used to robustly and parallelly estimate activation level features from all channels of unfiltered single-trial fNIRS data (Abdelnour and Huppert, 2009). For a given measurement channel, the transition equation and observation equation can be described as:


(2) $$\begin{array}{l} \{\begin{array}{c} \beta_{k}=\text{A}\beta_{k-1}+{\text{w}}_{k}\\ \Delta c_{k}={\text{X}}_{k}\beta_{k}+v_{k} \end{array} \end{array}$$

where $\beta ={\left[\beta_{k}^{1},\beta_{k}^{2},\ldots ,\beta_{k}^{L}\right]}^{T}\left(k=1,2,\ldots ,T\right)$ is the state vector representing the magnitude of each explanatory variable in the GLM estimated at time step *k*, Δ*c*_*k*_ is the measured raw Δ[HbO] signal at time step *k*. Since the magnitude of each explanatory variable is slowly varying with time, it can be assumed that the state **β**_*k*_ is a random walk with zero drift in the transition equation over time. Therefore, the state transition matrix **A** equals the identity matrix (Hu et al., 2010). As the random walk process is a non-stationary process, the state vector can be updated iteratively using KF. The distribution of process noise and observation noise are $w_{k}\sim \;N\left(0,\text{Q}\right)$ and $v_{k}\sim \;N\left(0,\text{R}\right)$, respectively. The *priori* estimates of the process noise covariance **Q** and the observation noise covariance **R** are set to **Q** = (1%)^2^**I** and **R** = (1.5)^2^**I** (Abdelnour and Huppert, 2009), where **I** is the identity matrix. The iterative process for updating the estimate of state **β**_*k*_ can be found in Hu et al. (2010). The state vector **β**_*k*_ is initialized to zero.

After processing the observed single-trial Δ[HbO] data through the iterative process, the **β**_*T*_ estimated at the last time step *T* is the final reconstructed **β**. The first element $\beta_{T}^{1}$ in the final estimated vector **β**_*T*_ is the estimated activation level, which represents the amplitude of the HRF. The $\beta =\left[{\left(\beta_{T}^{1}\right)}^{1},{\left(\beta_{T}^{1}\right)}^{2},\cdots \;,{\left(\beta_{T}^{1}\right)}^{D}\right]$ is a feature sample of the observed single-trial data, which consists of the estimated level features for all channels. ${\left(\beta_{T}^{1}\right)}^{d}\left(d=1,2,\cdots \;,D\right)$ denotes the level feature extracted from the *d*-th channel, and *D* is the total number of measurement channels.

### Adaptive Gaussian mixture model

The a-GMM is a well-known model for data clustering and classification. The uncertainty of the a-GMM parameters can be described by a probability distribution to form a hierarchical probability model. When a new sample arrives, variational Bayesian inference is used to update a-GMM parameters, using the previous parameter distributions as *priors*. Then, the a-GMM gives clustering labels to the new sample data points, and the clustering parameters are updated by a small amount of data because the *priors* are strong. The detailed derivation process of the unsupervised a-GMM approach can be found in the literature (Li et al., 2017), and here, we only give a brief description.

#### Probability model

For robustly extract level features by KF, the level feature sample $\hat{\beta }\in {\mathbb{R}}^{\text{1}\times D}$ extracted from single-trial fNIRS data for all channels from class *k* are modeled as having a multivariate normal distribution:


(3) $$\begin{array}{l} \hat{\beta }\sim \;\pi_{k}N\left(\mu_{k},\Lambda_{k}^{-1}\right) \end{array}$$

where π_*k*_(*k* = 1, 2, ⋯ , *K*) is the probability of the *k*-th category of fNIRS data in the a-GMM, *k* indicates the category of fNIRS data, and *K* is the total number of categories of fNIRS data. *K* is set to 2 in this study, but it has no limitations in a-GMM. **μ**_*k*_ is the *d*-dimensional mean vector, $\Lambda_{k}^{-1}$ is the *d* × *d* covariance matrix, and **Λ**_*k*_ is the precision matrix. To solve the parameters π_*k*_, **μ**_*k*_, and **Λ**_*k*_ in the Gaussian mixture distribution, first, the non-informative *prior* distribution needs to be selected. These *prior* distributions are generally determined by the conjugate distribution method, Jeffery principle, and principle of maximum entropy (Li et al., 2017). The non-informative *priors* are shown as follows:


(4) $$\begin{array}{l} \{\begin{array}{l} \pi_{k=1,\ldots ,K}\sim \;SymDir\left(j,\alpha_{0}\right)\\ \Lambda_{k=1,\ldots ,K}\sim \;W\left({\text{W}}_{0},v_{0}\right)\\ \mu_{k=1,\ldots ,K}\sim \;N\left[m_{0},{\left(\beta_{0}\Lambda_{k}\right)}^{-1}\right]\\ {\text{z}}_{n=1,..,N}\sim \;Mult\left(1,\pi \right) \end{array} \end{array}$$

where *N* is the total number of trials *SymDir*(·), is the *j*-dimensional symmetrical Dirichlet distribution, W(·) is the normal-Wishart distribution, *Mult*(·) is the multinomial distribution, and the **z**_*n*_ is the latent variable. For each observed feature sample ${\hat{\beta }}_{n}$, we have a corresponding latent variable **z**_*n*_ = {*z*_*n*1_, ⋯ , *z*_*nK*_} comprising a 1-of-*K* binary vector with elements *z*_*nk*_ for *k* = 1, …, *K*. The **z**_*n*_ is used to indicate the category to which the corresponding observed feature sample belongs to the fNIRS data. In order to distinguish the parameters of the joint probability distribution, the above-mentioned *prior* distribution parameters such as *K*, α_0_, β_0_, **W**_0_, *v*_0_, and *m*_0_ are known as hyper-parameters. During the parameter fitting, these hyper-parameters are fitted to the training data. An independent Gaussian-Wishart prior defined using known hyper-parameters can govern the random variable of **μ**_*k*_ and **Λ**_*k*_ of each Gaussian component, given by:


(5) $$\begin{array}{l} p\left(\mu_{k},\Lambda_{k}\right)=\prod_{k=1}^{k}N\left[\mu_{k}|m_{0},{\left(\beta_{0}\Lambda_{k}\right)}^{-1}\right]W\left(\Lambda_{k}|{\text{W}}_{0},v_{0}\right), \end{array}$$

where *m*_0_, β_0_, **W**_0_, and *v*_0_ are hyper-parameters in the *prior* distribution.

#### Variational Bayesian inference

The variational Bayesian method is used to classify new sample data and to update the hyper-parameters of the a-GMM approximately. Then, the Bayesian *posterior* of the parameters is calculated using the previous parameter distributions regarding *priors*. However, the form of the *posterior* probability is usually extremely complicated, and then we use the mean-field theory to find another simple model (inferred *posterior* distribution) to approximate instead of the true *posterior* distribution (Bishop, 2006). The difference between the *posterior* distribution and inferred *posterior* distribution is measured using the Kullback-Leibler (*KL*) divergence (Li et al., 2017).

The variational inference is to find the settings of the hyper-parameters which minimizes the *KL* divergence. This is equivalent to maximizing the variational lower bound. The process is performed in an iterative manner, assigning probabilistic labels to new sample data, and sequentially updating a-GMM hyper-parameters until the lower bound converges. The detailed iteration formula derivation can be found in (Bishop, 2006) and (Li et al., 2017). For the class labels, we define responsibility *r*_*nk*_ as the probability that the observed sample data ${\hat{\beta }}_{n}$ belongs to class *k*. We set *r*_*nk*_ as follows:


(6) $$\begin{array}{l} r_{nk}\propto \pi_{k}|\Lambda_{k}{|}^{1/2}\exp\left[-0.5{\left({\hat{\beta }}_{n}-\mu_{k}\right)}^{T}\Lambda_{k}\left({\hat{\beta }}_{n}-\mu_{k}\right)\right] \end{array}$$

The normalized *r*_*nk*_ satisfies the condition $\sum_{k=1}^{K}r_{nk}=1$. In the present study, convergence in the iterative algorithm is regarded as the change of the lower bound of < 0.1%. Besides, we also set a hard limit of 200 iterations. After the iterative algorithm ends, the class label for each new sample data *n* belongs to class *k* with the highest responsibility *r*_*nk*_. This is the maximum a *posteriori* estimation. After that, when more sample data are reached, these updated hyper-parameter values are used for the next run of the algorithm.

#### Transition model

The difference between the a-GMM approach and the GMM approach is the addition of a transition model, which is used to manage the hyper-parameters and, thus, change the parameters of the a-GMM (Li et al., 2017). Since we hope that the a-GMM classifier can adaptively track the changes of task-evoked brain activation patterns over time. This requires that the parameters of the a-GMM are updated with newly arrived sample data rather than becoming increasingly deterministic based on the cumulative statistics of the previous sample data (i.e., the parameter updates from newly arrived data will be smaller and smaller). To this end, we need to model the change in the parameters of the a-GMM classifier over time in order to handle the change process of the activation pattern adaptively.

The transition model governing the hyper-parameters of the *prior* distribution is presented in Equation (7). The transition model can effectively force a certain degree of forgetting of the old information so that the newly arrived data can still update the a-GMM parameters. It makes the center of the distribution of the a-GMM classifier parameters constant while the distribution becomes wider, i.e., reduces the certainty of the parameters. The parameters of the transition model are used to directly reflect the rate of change of the hyper-parameters, instead of simply using statistical variables to update. This allows the classifier can classify tasks adaptively over time. The hyper-parameters for the *k-*th category of fNIRS data are directly updated as follows:


(7) $$\begin{array}{l} \{\begin{array}{l} {\tilde{\beta }}_{0}^{k}=\beta_{0}^{k}/\left(1+c_{1}\beta_{0}^{k}\right)\\ {ṽ}_{0}^{k}=v_{0}^{k}c_{2}\\ {\tilde{\text{W}}}_{0}^{k}={\text{W}}_{0}^{k}/c_{2} \end{array} \end{array}$$

where 0 ≤ *c*_1_ and 0 < *c*_1_ ≤ 1 are constants indicating the rate of change of parameters. The right side of the equation is the posterior hyper-parameter value from the previous algorithm run, while the left side with the tilde sign is a *prior* hyper-parameter value for the next run. Variational inference is performed on one or a batch of new sample data by running the algorithm to converge.

## Experiments

### Simulation experiments

#### Simulation of fNIRS signal

A series of simulation experiments of random variations in the spatial patterns of brain activation over time is performed. First, we simulated task-related brain fNIRS signals (i.e., Δ[HbO]), then simulated task-evoked random variations in activation patterns of the prefrontal region over time, and finally performed adaptive decoding for two different tasks (i.e., MA vs. MS). The simulated fNIRS signal of one channel is modeled as a linear combination of five components (Duan et al., 2018):


(8) $$\begin{array}{l} {\text{s}}_{\text{fNIRS}}\;=\text{h}r\text{f}+\text{b}\text{p}+\text{b}\text{r}+\text{b}\text{l}+\text{r}\text{n} \end{array}$$

where **hrf** is the hemodynamic response of the task-evoked prefrontal regions, **bp** and **br** are changes in fNIRS signal induced by blood pressure and respiration, respectively, **bl** is the baseline drift, and **rn** is the random noise (systemic noise), i.e., additional background white noise during the measurement period of the instrument, and **s**_fNIRS_ is the final simulated fNIRS signal.

**Figure 1:**
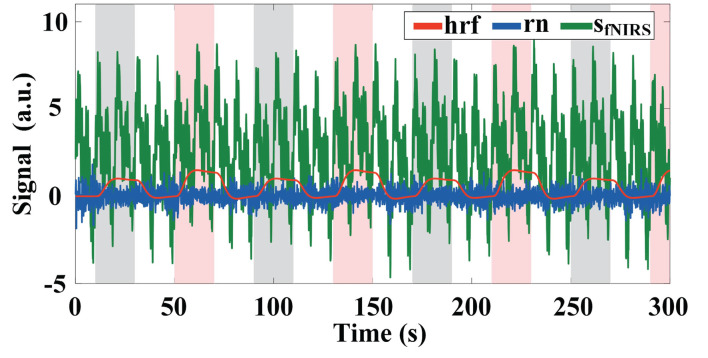
Example of simulated fNIRS signal with the amplitude of the baseline set to 2, the initial amplitude of the physiological interference set to 2, and a Gaussian white noise with *SNR* = 10 dB. The gray and pink highlighted rectangles indicate stimulation periods for task 1 and task 2, respectively.

The detailed settings for generating the simulated fNIRS signal are shown in Table 1. An example of the simulated **hrf**, **rn**, and the final combined fNIRS signal is illustrated in Figure 1. The time series of the **hrf** component is generated by convolving the canonical HRF (cHRF) evoked by a single stimulus with a boxcar function for task activation time series, representing the task and rest states alternation. The cHRF is modeled as a linear combination of two different gamma-variant time-dependent functions (Glover, 1999). The simulated **hrf**s evoked by the two different tasks (i.e., MA vs. MS) are normalized to ensure equal levels of activation. The time series of **hrf** components of all channels are then multiplied by a spatial filter that varies with time and obeys a two-dimensional (2D) Gaussian distribution to simulate dynamic changes in brain activation patterns. The time series of the physiological interference components are generated based on realistic a priori physiological parameters, and then multiplied by a spatial filter whose values for each channel are randomly generated from the standard normal distribution and are statically invariant, i.e., do not change over time (Li et al., 2017). The **bp** signal is composed of two sinusoids representing heartbeat (1.0 Hz) and Mayer wave (0.1 Hz). The **br** signal is a 0.2 Hz respiration signal (Scarpa et al., 2013; Hoang-Dung et al., 2018). The initial amplitudes of **bp** and **br** are set to 10, at which point the average *SNR* (**hrf** for signal and all noise components for noise) across all channels is −31.35 dB, i.e., the noise level is 35. This higher noise level is a more realistic simulation of the realistic measurement scenario. Each sinusoid has a random phase and amplitude distortions. The baseline amplitude is set to *bl* = 10.

**Table 1:** Detailed settings for generating simulated fNIRS signal.

	**hrf**	**bp**	**br**	**bl**	**rn**
Generation/parameters	cHRF^*^boxcar	Heartbeat (1.0 Hz)	Respiration	*bl* = 10	*SNR* = 10 dB
		Mayer wave (0.1 Hz)	(0.2 Hz)		
		Initial amplitude = 10	Initial amplitude = 10		
Spatial filter	Dynamic variation	Randomly generated and static	Randomly generated and static	-	-

* Denotes the convolution operator.

To more realistically mimic the measurement process, we also add Gaussian white noise (i.e., **rn**) with a signal-to-noise ratio (*SNR*) of 10 dB to the baseline for each channel. The difference in *SNR* between the $\bar{\text{hrf}}$ ($\bar{\text{hrf}}=\text{hrf}+\text{b}\text{l}$) and the baseline (**bl**) at time step *k* is proportional to the square root of the absolute values of the difference between the amplitude of the hemodynamic response $\bar{\text{hrf}}$ and the baseline (i.e., $\Delta SNR_{k}\;\infty \;\sqrt{\left|\bar{\text{h}\text{r}{\text{f}}_{k}}-bl\right|}$; Wang et al., 2020). $\bar{\text{hrf}}$ signal indicates **hrf** signal with baseline drift.

#### Simulation of randomly varying activation patterns

**Figure 2:**
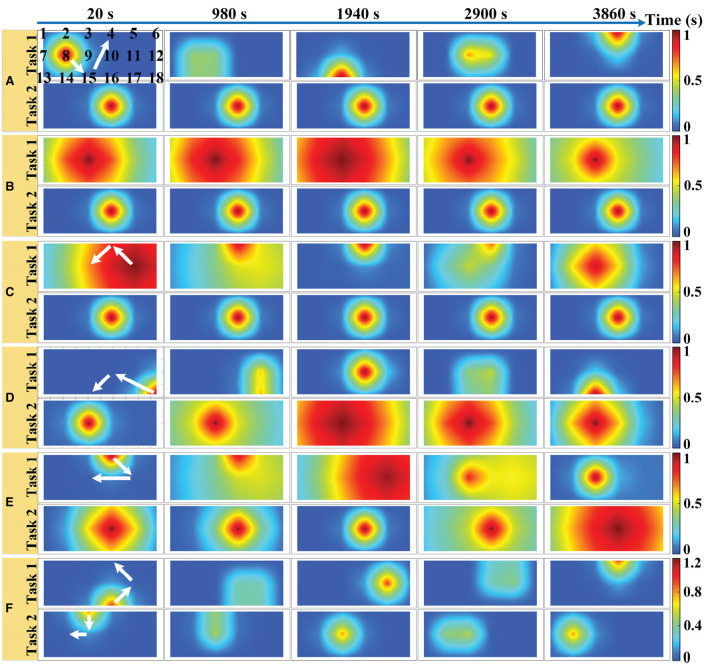
Six simulations with random varying spatial patterns of brain activation in prefrontal regions: **(A)** The center of the activation region is the random walk but its size remains constant. **(B)** The size of the activation region varies randomly but its center remains the same. **(C)** Both the center and the size of the activation region change randomly with time. **(D)** The center of the task 1 evoked activation region randomly walks while the size of task 2 evoked activation region varies randomly. **(E)** Both the center and the size of the task 1 evoked activation region change randomly with time while the size of task 2 evoked activation region varies randomly. **(F)** The center of the activation region is random walk for both tasks, but the amplitude of the HRF for task 1 is randomly varying, while that of task 2 remains constant. The white arrows indicate the direction of linear movement of the center of the activation region. The black numbers indicate the location of the sampling channels.

We simulated an 18-channel montage (3 × 6) of fNIRS measurements in the PFC region with a sampling rate of 10 Hz, as shown in Figure 2A, where the black numbers indicate the position of the channels. One trial of the simulated fNIRS data lasts 40 s, in which the first 20 s are the rest periods and the last 20 s contain the task-related activation. A total of 500 trials were simulated in one simulation, with alternating trials for task 1 and task 2. The simulated activation pattern varies randomly once every 50 trials and in total it varies randomly 10 times in one simulation. To the best of our knowledge, the randomly varying activation pattern has not been previously simulated in any fNIRS-BCI study. To mimic the possible changes more realistically in the spatial pattern of brain activation, a total of six simulations are implemented for randomly varying activation patterns as shown in Figure 2, which illustrates the optical topographies (OT) of random variations in activation patterns over the first 100 trials. Figures 2A–F correspond to simulation experiments 1–6, respectively.

For a random walk in the center of the activation region (Figure 2A), the center of the activation region moves linearly during the two different activation pattern changes as shown by the white arrow in Figure 2, but the change of the center of the activation region from the initial point to the end point is a random walking process. In simulation 1, the HRF spatial filter obeys a 2D Gaussian distribution with a covariance matrix of 0.2**I** (**I** denote the identity matrix) and remains constant, changing only its center. Two spatial filters are randomly generated from 2D Gaussian distributions with three different covariance matrixes (0.2**I**, 1.0**I**, and 5.0**I**, respectively) as the initial and final patterns of random variation and multiplied with the HRF of all channels to achieve random variation in the size of the activation region (Figure 2B). The change in the HRF spatial filter from the initial pattern to the final pattern is a linear and slow morphing in time, whose specific setup can be found in the literature (Li et al., 2017). The pattern variation in Figure 2C is a superimposed hybrid pattern of Figures 2A,B, i.e., both the center and the size of the activation region change randomly with time. When the size of the activation region evoked by task 2 in the simulations of Figures 2A,C no longer remains stationary also varies randomly, corresponding to the simulations of Figures 2D,E. The amplitude and waveform of the HRF are the same for all channels between the two tasks in simulations 1–5, and the amplitudes are normalized to 1 indicating the same level of activation for both tasks. Additionally, we also simulated differences in the amplitude of the hemodynamic responses evoked by two tasks, as shown in Figure 2F. The difference in the HRF amplitude for the two tasks was set to be small in the stochastic dynamic variation of the activation pattern. The HRF magnitude for task 2 is normalized to 1, whereas that for task 1 is randomly generated from a uniform distribution of 1.1–1.2 at each pattern change. The centers of the activation regions for both tasks are randomly walking rather than stationary, and the size of the activation regions remains constant during the random variation.

Since the feature extraction and classification methods for simulated experimental data and paradigm experimental data are the same, a detailed description of the feature extraction, feature data normalization and data classification for simulated experimental data can be found in Subsections Feature extraction and Classification of the “Paradigm experiments” section. To better evaluate the classification accuracy of the classifier for all simulated experiments, 10 runs of 10-fold cross-validation were performed.

### Paradigm experiments

#### Participants

Eight healthy right-handed participants (mean age: 23.5 ± 2.1 years, three men and five women) were recruited from the students at Tianjin University to conduct the experiments. None of the participants had reported a previous history of any psychiatric, neurological, or brain disorder. The study was conducted with informed consent and received ethical approval from Tianjin University.

#### Data acquisition

**Figure 3:**
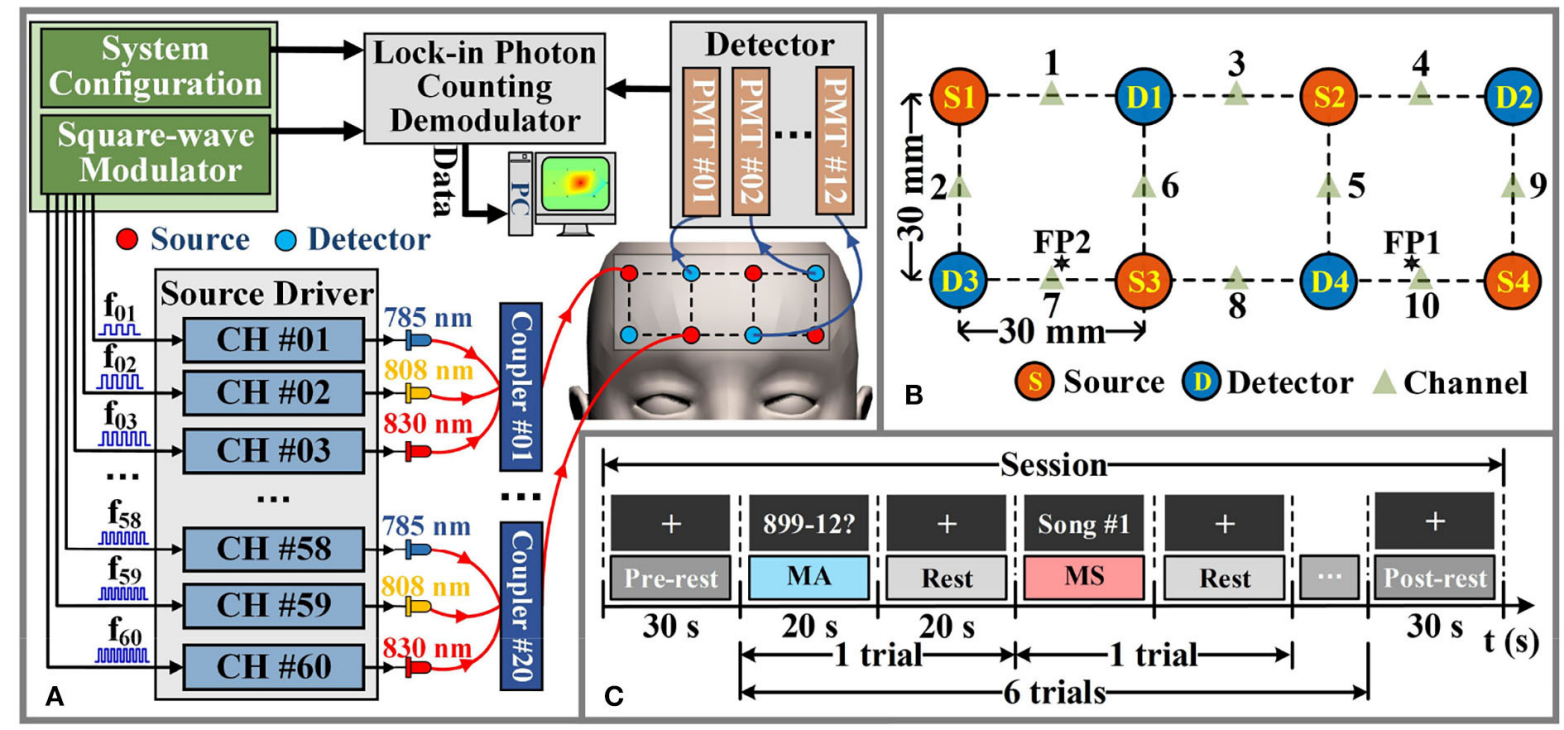
Hardware instruments and experimental paradigms for *in-vivo* experimental data acquisition. **(A)** The schematic diagram of the fNIRS-DOT instrument. **(B)** Experimental source and detector configuration. **(C)** Experimental paradigm of MA and MS.

We have implemented a continuous wave fNIRS diffuse optical tomography (DOT) system that adopts a lock-in photon-counting technology to enable multi-channel parallel measurements, as exhibited in Figure 3A (Liu et al., 2019). We use a total of four source-pairs, each containing both 785 nm and 830 nm laser diode sources and four photomultiplier (PMT) detectors form a single-lattice arrangement scheme (Figure 3B), resulting in 20 measurement channels for collecting fNIRS signals, are secured against the PFC region of the participant. The system sampling rate for data acquisition is 4 Hz, which is lower than the setting of the simulation experiment. However, it is a moderate temporal resolution for our measurement system, which can improve the *SNR* of the measured data and meet the requirements of real-time data processing.

According to the international 10–20 system, the source-pair and detector are arranged to cover the optode positioning points FP1 and FP2 of the PFC region, as shown in Figure 3B. In the given configuration, we only considered signals arising from the source-detector distance of 30 mm (Power et al., 2010).

#### Experimental protocol

In the paradigm experiment, all participants performed the MA and MS mental tasks. The MA is one of the most widely used and robust mental tasks in fNIRS-BCI research (Power et al., 2012). The mental workload level of the MA task was the same because participants were asked to repeatedly subtract a small number (between 9 and 15) from a randomly generated three-digit number. For the MS mental task (Hwang et al., 2016), participants silently rehearsed self-selected Chinese song fragments that they felt would provoke a strong and positive emotional response within them. This means that the MS task utilizes the emotional component of the music. The self-selected Chinese songs were different in each trial for each participant. The self-selected song presented in each trial was randomly generated from the participant's self-constructed song library.

The schematic diagram of the *in-vivo* experimental paradigm is shown in Figure 3C. Each participant was required to perform 20 data collection sessions on the same day. Figure 3C illustrates a data collection session consisting of a 30 s pre-rest period (i.e., baseline period), 6 trials, where each trial consisted of a 20 s of MA or MS mental task followed by 20 s of rest, and a 30 s post-rest period in the end. During each data collection session, each participant performed 6 trials in which MA trials and MS trials were alternated. The fNIRS optodes were not removed from the participants during all data collection sessions. Data collection for all participants was completed over a period of 5 days. The total number of trials collected for each category of the mental task for each participant is 60. Therefore, the total number of trials collected for each participant is 120.

#### Signal pre-processing

The 5th order zero-phase Butterworth digital low-pass filter with a cut-off frequency of 0.5 Hz is used to eliminate the heartbeat noise and high-frequency instrument noise for the raw light intensity data. Then, the coefficient of variation (*CV*) of the low-pass filtered raw light intensity signal is calculated to evaluate the quality of data (i.e., the effect of motion artifacts on the measured data) for each measurement channel. The *CV* can be defined as:


(9) $$\begin{array}{l} CV=\left(\sigma \left[\text{I}\right]/E\left[\text{I}\right]\right)\times 100 \end{array}$$

where **I** denote the raw light intensity for a data collection session. *E*[·] and σ[·] denote the mean and standard deviation, respectively. When the measured raw light intensity data meets the condition of *CV* > 10 (Piper et al., 2014), the channels are rejected and not used in the subsequent further data analysis. Furthermore, we used the modified Beer-Lambert law (MBLL) to convert the raw light intensity signal into Δ[HbO] and Δ[HbR] (Weyand et al., 2015). The unfiltered Δ[HbO] signal, which has not been subjected to BPF or other filtering methods to remove baseline drift and global physiological interferences, was used in the GLM-KF for adaptive extraction of level features. The GLM considers baseline drift and physiological interferences when modeling the fNIRS signal. However, when applying the GLM-KF method to real fNIRS data, it is required to consider pre-task calibration. In long-term measurements of real fNIRS data, excessive cognitive load can cause rapid fatigue of the subject, causing changes in baseline across data collection sessions for the same subject. In addition, due to individual differences, the baselines between subjects were also different. Therefore, the pre-task calibration for each session of each subject used the fNIRS data for the baseline period under that session. The parameter settings of the BPF for the extraction of statistical-features are described in detail in Section Hemodynamic changes. A zero-phase digital high-pass filter (0.018 Hz cutoff) in the BPF effectively removes the baseline drift when extracting statistical-features (Bejm et al., 2019).

#### Feature extraction

In the previous fNIRS-BCI study, the most widely used features including mean, variance, slope, skewness, and kurtosis, which these BPF-statistical features are not designed to better characterize hemodynamic responses (Weyand et al., 2015; Hong et al., 2020). The fNIRS-BCI desires adaptive extraction of dedicated level features of single-trial fNIRS data. In this study, KF was used to extract the activation level recursively by solving the GLM.

**Figure 4:**
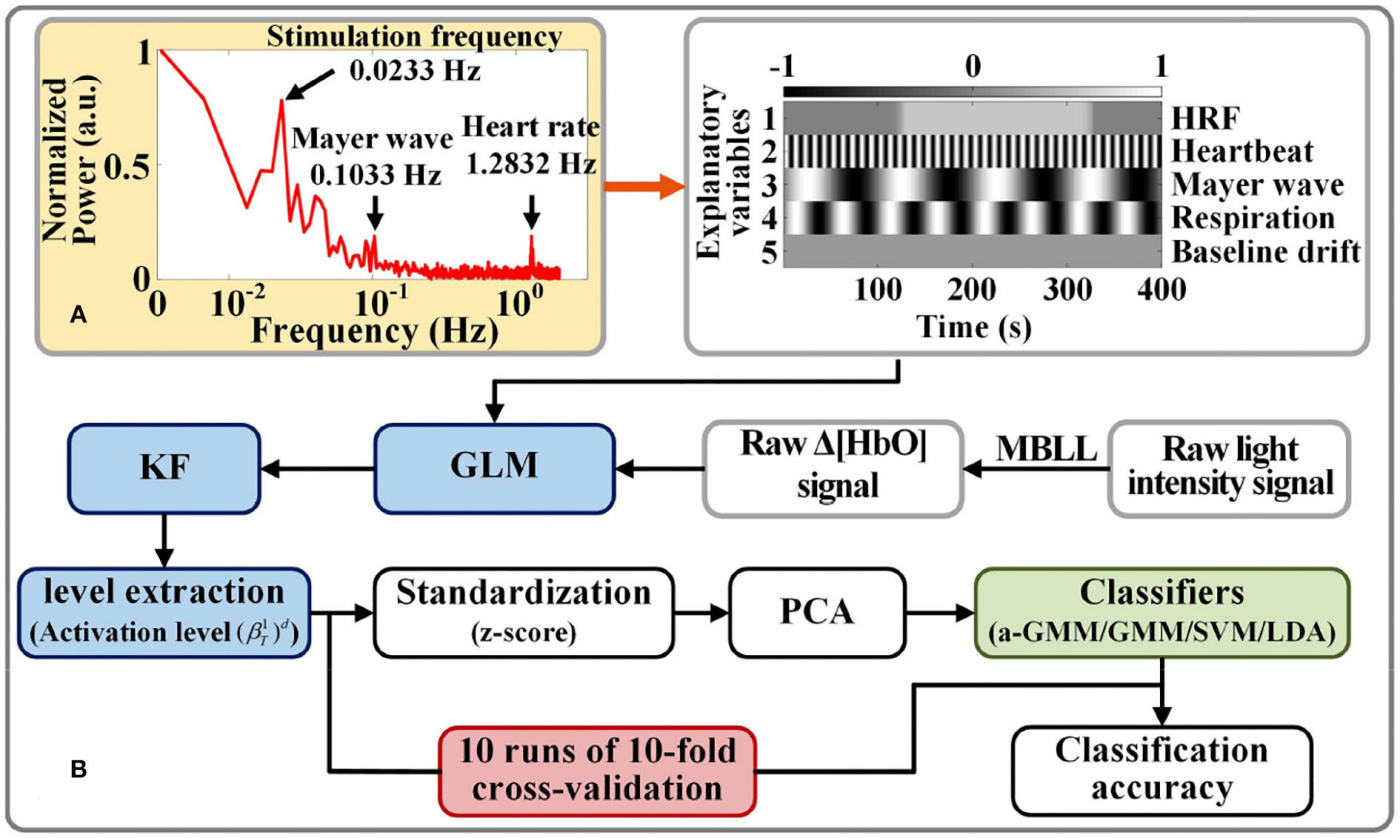
Activation level feature extraction and classification strategy for fNIRS signals: **(A)** Example of a spectrogram of participant 1 for frequency selection of physiological interferences in the design matrix. **(B)** Flow chart of fNIRS signal classification based on level features.

The frequency selection for each physiological interference of the design matrix in GLM is based on the fast Fourier transform of the raw light intensity measured by each participant for spectrum analysis. Figure 4A shows the frequency spectra for participant 1, where the normalized power amplitude of the stimulation frequency (0.0233 Hz) is relatively higher, and it is slightly less than the theoretical value (1/40 = 0.0250 Hz) of the frequency of neural activation. This is due to the hemodynamic response being delayed by 2–3 s after the neural activity (Tomita et al., 2014).

#### Classification

The assessment metric used to quantitatively assess the classifier's ability to accurately discriminate the task is accuracy, defined as shown below (Wickramaratne and Mahmud, 2021).


(10) $$\begin{array}{l} Accuracy=\frac{TP+TN}{TP+FP+TN+FN} \end{array}$$

where *TP* is the number of true positives, *FP* is the number of false positives, *TN* is the number of true negatives, and *FN* is the number of false negatives. The accuracy results, in this paper, are all obtained from the test set data. The classification process is performed in an offline mode. To better assess the classification performance of the classifier for the MA and MS mental tasks, 10 runs of 10-fold cross-validation were also performed as in the simulations.

We compared the classification performance of the proposed KF/a-GMM approach with the BPF-statistical features based classical methods such as the GMM classifier, which uses GMM to fit parameters on the same training data in the same way, but does not update the parameters when it classifies the testing data, SVM classifier with radial basis kernel function and regularization parameter of 20, and LDA classifier. The flow chart of activation level-based fNIRS signals decoding is shown in Figure 4B. Feature samples are normalized by calculating z-scores. For these normalized values, we use the principal component analysis (PCA) method with the smallest principal component number whose cumulative contribution rate exceeds 95% to reduce the dimensionality of feature sample data (Li et al., 2020). The final dimensionality reduction data is used to construct a classifier.

#### Statistical analysis

In the real-time classification of mental tasks, we are also more interested in whether the channel is significantly activated at each time step *k*. First, we proposed the null hypothesis ($H_{0}:{\text{c}}^{T}\beta_{k}=0$) that the channel is not activated at time step *k*, where **c** is a vector of contrast used to select the coefficients of interest. This hypothesis is tested by calculating the relevant *t*-values from the estimated GLM coefficient vectors at all time steps and then performing a *t*-test with significance criteria of 0.05 (Hu et al., 2010). Finally, we used paired-samples *t*-tests of SPSS 22.0 software (IBM SPSS Inc., Chicago, IL, USA) with significance criteria of 0.05 for statistical analysis of classification accuracy.

## Results

### Simulative investigations

#### Extraction of activation level feature

**Figure 5:**
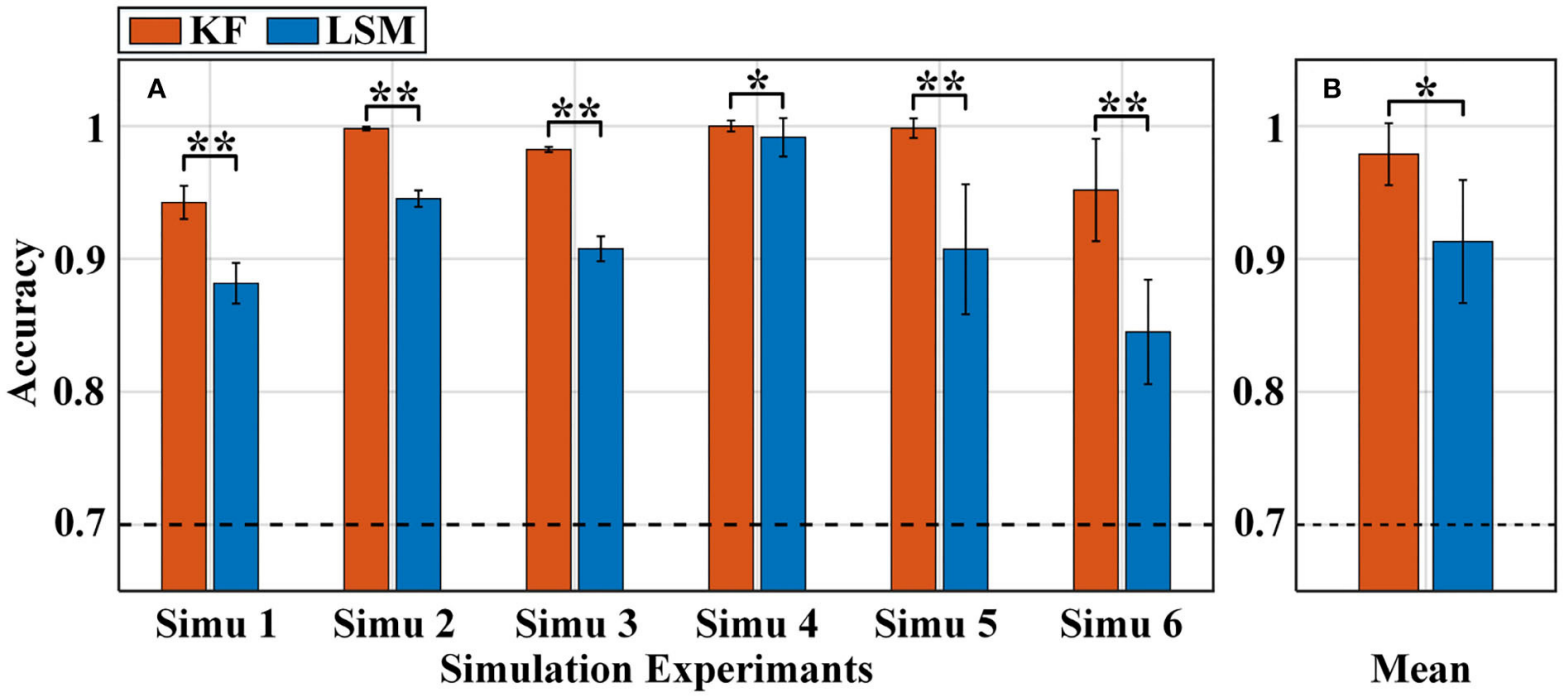
Classification accuracy of the a-GMM classifier in all simulations was based on the level feature extracted by KF and LSM methods. **(A)** Average accuracy of each simulated experiment, **(B)** average accuracy across all simulated experiments. The black dashed line indicates 70% accuracy of effective binary BCI communication. Error bars indicate the standard deviations, *Represents the significant difference, *p < 0.01 and **p < 0.001.

Both the KF and LSM can extract the activation level features for each channel of the measurement data. We construct the same a-GMM classifier based on these two methods to extract activation level features, respectively. Then, the effect of both level extraction methods on accuracy in all simulations is shown in Figure 5. Figure 5A shows the average accuracy of 10 runs of the 10-fold cross-validation for each simulation experiment. The average accuracy based on the two different level extraction methods across all simulations is illustrated in Figure 5B.

As observed from the results that the accuracy of the KF-based levels was higher than that of the LSM-based levels in each of the simulations (Figure 5A). The average accuracy obtained based on KF-level was 97.89% higher than that based on LSM-level at 91.31% across all simulations (Figure 5B). The paired samples *t*-test yielded statistically significant differences in the two methods of level extraction. Since both KF and LSM invisibly encompass the filtering process when solving the GLM, the filtering effect of KF is better than that of LSM, resulting in a more accurate estimation of activation levels. Therefore, KF was used for the level feature extraction method in the subsequent sections.

#### Recognition of randomly varying activation patterns

**Figure 6:**
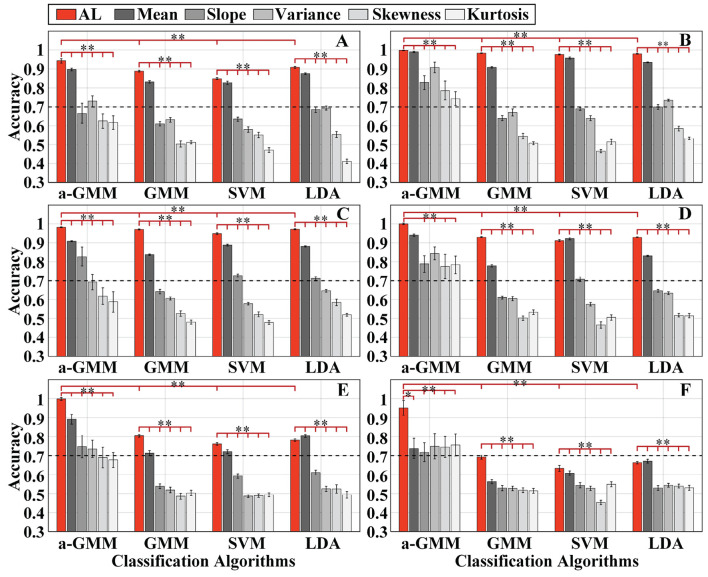
The single-trial average accuracy of six simulations with randomly varying activation patterns of the brain under Gaussian white noise with *SNR* = 10 dB added at the baseline: **(A–F)** Are the accuracy results of simulations 1–6, respectively. AL is an abbreviated form of activation level. The red bars indicate accuracy based on the activation level features, while the gray bars with different saturation levels indicate the accuracy based on five BPF-statistical features, namely mean, slope, variance, skewness, and kurtosis. Error bars indicate the standard deviations, *Represents the significant difference, empty: *p* > 0.05, *p < 0.01, and **p < 0.001.

The single-trial average accuracy of six simulations with randomly varying activation patterns under Gaussian white noise with *SNR* = 10 dB added at the baseline is illustrated in Figure 6. Figures 6A–F shows the results of the average accuracy of simulations 1–6, respectively. The results exhibit the accuracies obtained by the four classifiers based on activation level features and BPF-statistical features. For the same classifier, we only compared the accuracy based on the proposed level features with that based on the individual BPF-statistical features. This is because it would be fairer to compare the proposed level features with individual BPF-statistical features (for a one-to-one comparison) than with a set of BPF-statistical features (for a one-to-many comparison).

As observed from the accuracy results, the decoding accuracy based on level features was mostly higher than that based on BPF-statistical features in each classifier for each simulation. The accuracy of the level-based SVM classifier for simulation 4 was slightly lower than that of the mean-based one, and the accuracy of the LDA classifier for simulation 5 and simulation 6 performs in the same way. In addition, the paired-samples *t*-tests yielded significant differences in the level features and each BPF-statistical feature of each classifier in each simulation, except for the level feature vs. the mean feature for the a-GMM classifier in simulation 4. Moreover, the classification performance based on the mean feature was the best among the five BPF-statistical features. Moreover, the accuracy of a-GMM was mostly higher than that of GMM, SVM, and LDA in six simulations, whether based on BPF-statistical features or level features. Paired-samples *t*-tests yielded significant differences between a-GMM classifiers based on level features and other classifiers based on level features in all simulations.

As observed from the results, the KF/a-GMM approach can indeed accurately capture the random walk of the center of the activation region (Figure 6A), the random variation in the size of the activation region (Figure 6B), and their superimposed hybrid pattern (Figure 6C), with corresponding accuracies of 94.25, 99.81, and 98.25%, respectively. When the activation pattern evoked by task 2 changes from a stationary invariant state to a random variation as well, the accuracies of the control methods (relative to the KF/a-GMM) mostly decrease as illustrated in Figures 6D,E. It is also observed from the classification results that the accuracies of the control methods in simulation 5 were lower than that in simulation 4. However, the KF/a-GMM approach in simulations 4 and 5 still maintains a significantly higher accuracy of 99.98 and 99.85%, respectively. The accuracy of random varies magnitude of the HRF under a random walk in the center of the activation region was presented in Figure 6F. The accuracies of GMM, SVM, and LDA classifiers were all below 70%, while accuracies of a-GMM were all above 70%, whereas those based on BPF-statistical features are slightly above 70% and those based on level features have a significant advantage of up to 95.18%.

#### Noise sensitivity

To investigate the sensitivity of the KF/a-GMM method to noise, we added different levels of Gaussian white noise with *SNRs* of 1, 5, 10, 15, 20, 30, and 40 dB to the baseline for all channels of the simulated data. In the pre-processing of the fNIRS data, since the classical BPF method can effectively suppress most of the physiological interferences, the white noise in the passband of the filter cannot be eliminated. Therefore, the effect of different levels of Gaussian white noise on the classification results is investigated in all simulations with a fixed level of physiological interference.

**Figure 7:**
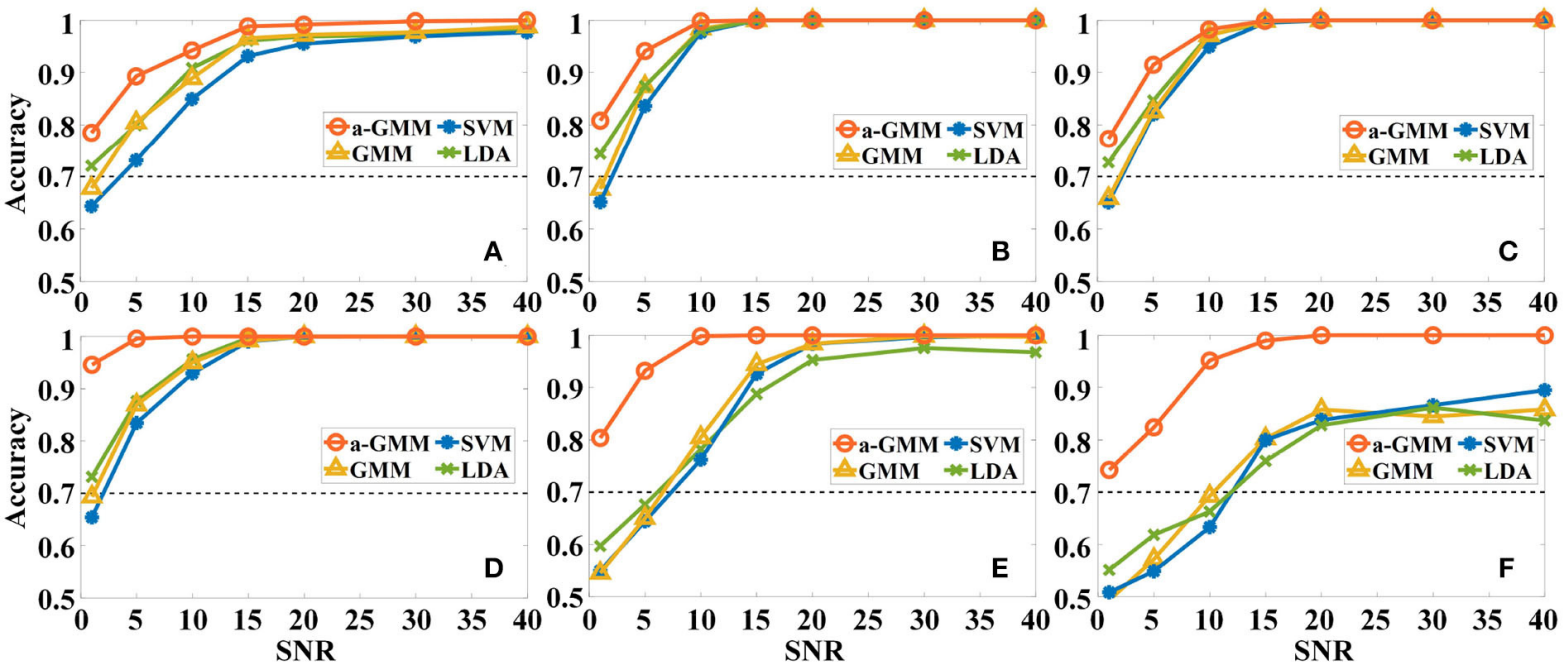
The effect of different levels of Gaussian white noise on the classification accuracies of the four classifiers based on the level feature. **(A–F)** Correspond to the accuracy results of simulations 1–6.

First, the effect of different levels of Gaussian white noise on the accuracies of the four classifiers based on the same level features was analyzed. As expected, the accuracy of all classifiers increases with *SNR* in the six simulations, as illustrated in Figure 7. However, the accuracy of a-GMM at each *SNR* was higher than the other three classifiers. Moreover, at lower *SNR* (*SNR* ≤ 10 dB), the a-GMM has a prominent decoding advantage in simulations 4–6. Even at the lowest *SNR* = 1 dB, the accuracy of a-GMM for all simulations was higher than 70%, which demonstrates superior noise robustness. In simulation 6 with random variation in magnitude of the HRF, the accuracy obtained by a-GMM demonstrated an overwhelming advantage compared to control classifiers.

**Figure 8:**
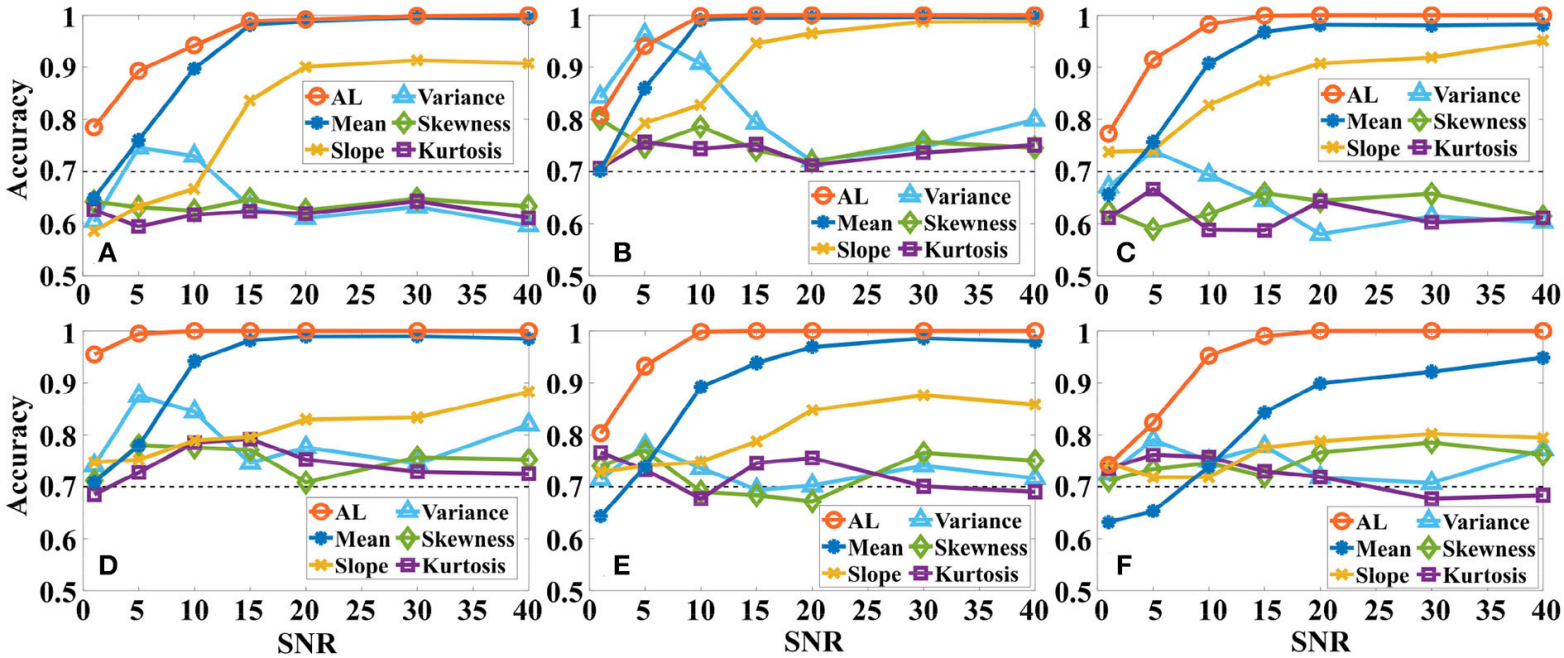
The effect of different levels of Gaussian white noise on the classification accuracy of the same a-GMM classifier based on level feature and BPF-statistical features. **(A–F)** Correspond to the accuracy results of simulations 1–6. AL is an abbreviated form of activation level.

In addition, we also analyze the effect of different levels of Gaussian white noise on the accuracy of the same a-GMM classifier based on level feature and BPF-statistical features as shown in Figure 8. As observed from the results, the accuracy of a-GMM based on activation level, mean, and slope features increases with *SNR* in all simulations, but the variance, skewness, and kurtosis features do not reveal any regularity. The accuracy of the skewness and kurtosis features fluctuated irregularly with the *SNR*, but both have lower accuracy at each *SNR* compared to the level feature. Also, an attractive phenomenon found in the accuracy results is that there is an abnormal fluctuation in the accuracy based on the variance feature in the lower *SNR* region, especially in simulation 2 where the fluctuation of *SNR* = 5 dB reaches a maximum peak of 96.25%. A reasonable reason for this occurrence may be that the white noise in the passband of the Butterworth BPF is not entirely suppressed, and the spurious activation caused by the white noise at this level has the strongest effect on the statistical characteristic-based variance feature, which happens to magnify the difference between the activation patterns of the two-class tasks and finally leads to abnormally high accuracy. However, the accuracy of the a-GMM based on the level feature was the highest among all simulations, except for the variance-based feature of simulation 2. In addition, the classification performance of the mean-based feature was the best among all BPF-statistical features when the *SNR* was >10 dB.

### *In-vivo* paradigm experiments

#### Hemodynamic changes

**Figure 9:**
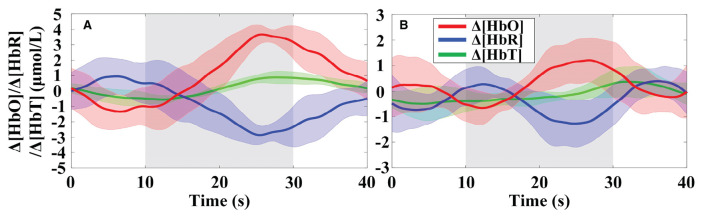
Trial-averaged hemoglobin concentration changes for participant 1 in the most significantly activated channels: **(A)** Channel #5 in the MA and **(B)** channel #6 in the MS were significantly activated. The gray highlighted rectangles indicate the stimulation periods. Δ[HbT] indicates the change in total hemoglobin concentration. The shades of the same color around the curves represent the standard deviation.

The primitive Δ[HbO] and Δ[HbR] data are band-pass filtered with the 5th order zero-phase Butterworth filter with cutoffs of 0.018 and 0.3 Hz to eliminate global physiological interference. The hemodynamic signals obtained across the six repeated trials of the MA and MS mental tasks during the two data collection sessions were averaged, respectively. To evaluate whether the channels are activated at each time step, the estimated GLM coefficients are converted into the corresponding *t*-statistics value. At time step *k*, a larger *t*-value for a channel indicates a more significant activation of that channel. The *t*-values for all channels of participant 1 after 15 s from the onset of the MA and MS tasks stimulation are presented in Table 2. As observed from the results in Table 2, the most significantly activated channels were channel #5 of the MA and channel #6 of the MS. The corresponding hemodynamic changes of the two channels were shown in Figure 9. As expected, an increase in Δ[HbO] and a decrease in Δ[HbR] were observed during the MA and the MS stimulation period as shown in the gray shaded area in Figures 9A,B.

**Table 2:** The *t*-values for all channels of participant 1 after 15 s from the onset of the MA and MS tasks stimulation.

	***t*** **-values**									
**Mental task**	**CH #1**	**CH #2**	**CH #3**	**CH #4**	**CH #5**	**CH #6**	**CH #7**	**CH #8**	**CH #9**	**CH #10**
MA	2.63	−8.05	−4.15	1.95	10.16	2.59	4.72	3.53	−14.15	−15.54
MS	−0.18	−1.27	−1.74	−1.00	−0.31	1.44	0.70	−0.07	−1.33	1.38

CH, channel.

**Figure 10:**
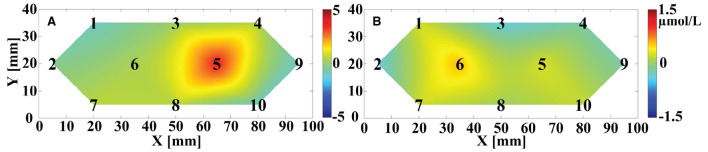
The OT of averaged Δ[HbO] across the six repeated trials for participant 1 evoked by **(A)** MA and **(B)** MS after 15 s from the onset of the task. The black numbers denote the position of the sampling channels. The vertical color bar indicates the range in μmol/L.

The OT of trial-averaged Δ[HbO] signal across 6 repetitions of MA and MS tasks during two data collection sessions for participant 1, after 15 s from the task onset, are shown in Figures 10A,B, respectively. The magnitude of the MA-evoked Δ[HbO] is significantly higher than that of the MS for participant 1. Furthermore, channels #5 and #6 were significantly activated under the MA and MS stimulation task, respectively.

#### Classification accuracy

**Figure 11:**
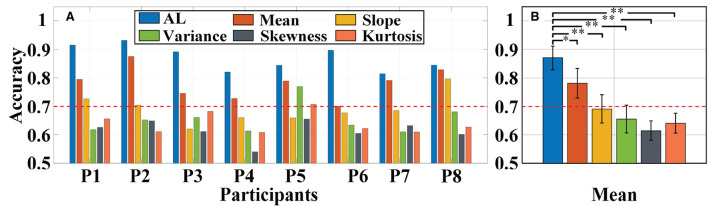
Classification accuracy of MA vs. MS obtained using the same a-GMM classifier based on different features extracted from single-trial data of all participants: **(A)** Individual accuracy and **(B)** average accuracy. Error bars indicate the standard deviations, *Represents the significant difference, *p < 0.01 and **p < 0.001. The red dashed line indicates 70% classification accuracy of effective binary BCI communication.

Primarily, we used the same a-GMM classifier to classify MA and MS tasks, comparing the single-trial accuracy between the level feature and the BPF-statistical features. The individual and average accuracy of MA vs. MS is illustrated in Figure 11. The red dashed line indicates 70% accuracy of effective binary BCI communication (Vidaurre and Blankertz, 2010). The paired-sample *t*-test yielded significant differences between activation level features and BPF-statistical features at average accuracy for all participants. As observed from the results, the individual and average accuracy obtained by the KF/a-GMM method are significantly higher than those BPF-statistical features. In addition, the average accuracy of only the activation level and mean feature reached above 70%, which has been regarded as a threshold for practical binary communication.

**Figure 12:**
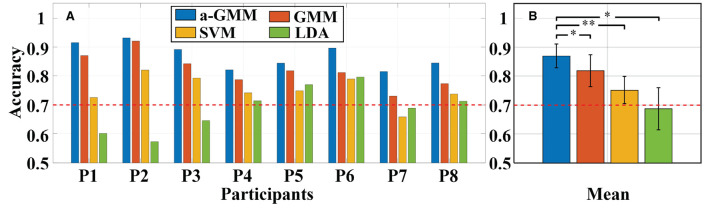
Classification accuracy of MA vs. MS for different classifiers constructed by extracting the same level features: **(A)** Individual accuracy and **(B)** average accuracy. Error bars indicate the standard deviations, *Represents the significant difference, *p < 0.01 and **p < 0.001. The red dashed line indicates 70% classification accuracy of effective binary BCI communication.

Then, different classifiers are performed to classify MA vs. MS based on the same level features. The individual and average accuracy of MA vs. MS is illustrated in Figure 12. The paired-samples *t*-test yielded significant differences between the a-GMM classifier and other classifiers in accuracy for all participants. As observed from the results, the average accuracy obtained by the a-GMM classifier was the highest among all classifiers, with an average accuracy of 87.01 ± 4.11%. However, only the LDA classifier obtained an average accuracy below 70% among all classifiers. For the a-GMM classifier, participant 1 and participant 2 achieved excellent classification accuracies of 91.50 and 93.09%, while participant 3 and participant 6 are also very close to 90%.

## Discussions

For *in-vivo* paradigm experiments, we initially compared the accuracy of the same a-GMM classifier based on different features, and the average accuracy of the KF/a-GMM approach reached 87.01 ± 4.11% for all participants, as shown in Figure 11. Compared with the skewness feature (61.50 ± 3.41%, *p* = 8.8975 × 10^−7^), the classification performance based on the level feature has a maximum improvement of up to 41.48%, and the minimum improvement is 11.31% compared with the mean feature (78.17 ± 5.22%, *p* = 0.0051). Then the classification performance of different classifiers based on the same level feature is compared. As observed in the results of Figure 12, up to 6.20, 15.72, and 26.50% improvement in average accuracy was achieved by a-GMM compared with GMM (81.93 ± 5.54%, *p* = 0.0012), SVM (75.19 ± 4.69%, *p* = 3.9656 × 10^−5^), and LDA (68.78 ± 7.26%, *p* = 0.0020) classifier for all participants, respectively. In classifying MA vs. MS, the classification accuracy of our proposed KF/a-GMM method was 12.71% improved over that of the light intensity based HMM classifier used by Power et al. (2010). Therefore, the KF/a-GMM approach can indeed enhance the accuracy of binary classification for MA and MS mental tasks.

**Figure 13:**
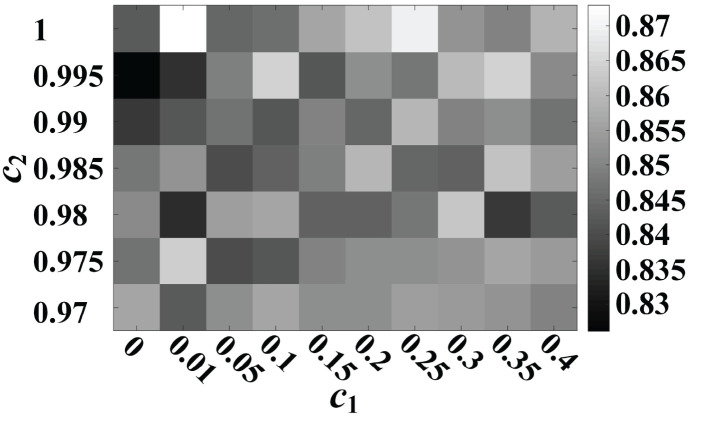
Effect of *c*_1_ and *c*_2_ parameters of the transition model on the 10-fold cross-validated accuracy of MA vs. MS for all participants.

The effect of the *c*_1_ and *c*_2_ parameters of the transition model in the a-GMM classifier on the 10-fold cross-validated accuracy of MA and MS for all participants is shown in Figure 13. The accuracy of a-GMM was the highest at *c*_1_ = 0.01 and *c*_2_ = 1. The *c*_1_ parameter adjusts the rate of change of the activation mean, slightly >0 will achieve higher accuracy. The *c*_2_ parameter is related to the rate of change of the activations covariance, and accuracy is probably better when it is close to 1. Further study on parameter selection can be adaptively iterated over a range of a priori parameters to obtain the optimal combination rather than relying on empirical selections.

Our proposed KF/a-GMM classification method has achieved considerable success in subject-level decoding. However, achieving the goal of across-subject fNIRS decoding is still quite challenging as the large individual differences make it more difficult to find population-level regularities that hold between individuals. Across-subject decoding in fNIRS-BCI classification is an important future direction (Raizada and Connolly, 2012; Jin and Kim, 2020). The drawback of *k*-fold cross-validation used to evaluate the classification model is that fNIRS data from the same subject are present in both the training and test datasets. Hence, classification models with parameters and hyper-parameters learned from the same subject's data may struggle to generalize to new subjects. Thus, the leave-one-subject-out cross-validation was used to evaluate and compare the performance of the classification model (Gholamiangonabadi et al., 2020). In each cross-validation iteration, one subject's data is used for testing and the remaining subjects' data for training to conduct the subject-independent evaluation. In the across-subject fNIRS decoding MA vs. MS, the decoding accuracy of the a-GMM classifier based on the same level features was 78.44%, higher than that of the GMM (52.81%), SVM (49.69%), and LDA (51.46%) classifiers. The preliminary research results showed that our proposed KF/a-GMM method can learn population-level regularities under the same mental task and has potential advantages in across-subject decoding. The across-subject fNIRS decoding mental task is more complex and needs further study in the future.

The levels estimated by the KF algorithm at each time point *k* can also be used as real-time features, which can subsequently be used for the real-time classification of the classifier. The potential advantage of KF lies in the real-time and parallel estimation of level features for all channels. The KF algorithm takes an average time of 0.0040 ± 0.0017 s (about 250 Hz) to estimate the simulated fNIRS signal with 18-channel at each time step in parallel on a computer configured with an Intel(R) Core (TM) i7-4790 CPU ^@^ 3.60 GHz and 16.0G RAM. In addition, the average time required by the a-GMM algorithm to decode each test data is 0.0035 ± 0.0023 s (about 286 Hz). Hence, both the KF and a-GMM have low computational costs when they deal with fNIRS data acquired at sampling rates of a few Hz to tens of Hz, and their fast computational speed greatly meets the requirements for real-time classification. It also incorporates the fact that a-GMM can be used for multi-class task classification (without the need to convert a multi-class classification problem into multiple binary classification problems), so that the KF/a-GMM approach can handle the real-time classification of multi-class tasks and be applied to long-term neurofeedback training (NFT) (Luhrs and Goebel, 2017) and rehabilitation training (Matarasso et al., 2021). The threshold for the a-GMM in the practical real-time BCI decoding is typically set to 50% (Abdelnour and Huppert, 2009). The KF/a-GMM method is used to calculate the estimation of the mental task at each time point after stimulus onset. If more than half of the time points within the stimulus period during a single-trial epoch are correctly classified, we obtain an overall correct classification label.

This study has some limitations that will be addressed in the future study. First, the baseline component added to the simulated fNIRS signal is a constant. It might be more realistic to give the baseline a Gaussian pattern. Second, since we use the HRF with fixed parameters in the design matrix, but the HRF of each participant is different, we should accurately estimate the HRF for each participant. Third, the physiological interference components of the design matrix can be acquired in real-time by auxiliary measurements, replacing manual selection based on spectral analysis. Fourth, as motion artifacts and probe registration have a large impact on single-trial level analysis, it is also worthwhile to investigate how to ensure the robustness of the single-trial GLM model. Measurement equipment for more accurate and rapid positioning of optodes and advanced algorithms for removing motion artifacts are also important directions for future study. Fifth, the initial values of the **Q** and **R** in the KF have a significant influence on the estimated results (Hu et al., 2010). However, the selection of **Q** and **R** based on prior knowledge at the beginning of the experiment also depends on the rich user experience to give an appropriate tracking of the evoked signals. Sixth, the combination of the proposed level features and BPF-statistical features to form a feature set is interesting and valuable and may be helpful in further improving the recognition accuracy of our proposed approach for single-trial mental tasks. Seventh, the comparison of classification methods in this paper is incomplete, comparing only the commonly used LDA classifiers and not the regularized LDA classifier (Bauernfeind et al., 2014) and the adaptive LDA classifier (Li et al., 2017). Eighth, considering the low sample size, preliminary conclusions have been drawn for now. Future studies will increase the sample size to corroborate again. Ninth, channel selection plays a critical role in classifying mental tasks for fNIRS-BCI by reducing data dimensionality, saving model training time, and improving model classification performance (Gulraiz et al., 2022). Besides the commonly used Fisher score method for channel selection (Hwang et al., 2016), the least absolute shrinkage and selection operator homotopy-based sparse representation method proposed by Gulraiz et al. for channel selection can improve the accuracy of walking and resting states (Gulraiz et al., 2022). This method has aroused our great interest and may improve the accuracy of our proposed KF/a-GMM. Tenth, the ongoing activation patterns may vary during the NFT session due to learning effects, and this process may affect the performance of brain state classifiers trained using data obtained before the session (Bagarinao et al., 2020). However, the biggest advantage of our proposed KF/a-GMM is that it is good at tracking changes in activation patterns, which can improve the accuracy of real-time brain states in NFT. On the other hand, the findings of Wang et al. demonstrated that visual-haptic NFT based on EEG-BCI improved cortical activation and the accuracy of MI (Wang et al., 2019). Thus, both our proposed KF/a-GMM and NFT can improve the classification performance of the BCI system, and jointly they can be used to improve cognitive function and enhance the quality of life of patients suffering from brain cognitive disorders. Finally, this approach currently only uses offline mode for data processing and analysis, and we will develop an online version.

## Conclusion

A joint adaptive classification approach KF/a-GMM that combines the KF and the unsupervised a-GMM classifier is proposed for accuracy-enhanced pattern recognition for mental tasks. Its effectiveness and advantages are validated by performing both simulation experiments and *in-vivo* paradigm experiments. The results show that this approach is an effective strategy for tracking random variations in brain activation patterns with time evoked by two-class mental tasks and considerably ameliorates the single-trial accuracy of unfiltered and unlabeled fNIRS data from MA and MS mental tasks. The average accuracy of the KF/a-GMM method for all participants in the MA and MS mental tasks was 87.01%, higher than the BPF-statistical features based GMM, SVM, and LDA classifiers. Overall, these results are encouraging and demonstrate the potential of the KF/a-GMM method to improve classification accuracy on mental tasks and provide a new perspective for real-time multi-class mental task classification.

## Data availability statement

The original contributions presented in the study are included in the article/supplementary material, further inquiries can be directed to the corresponding author/s.

## Ethics statement

The studies involving human participants were reviewed and approved by Tianjin University. The patients/participants provided their written informed consent to participate in this study.

## Author contributions

YZ: study design and article writing. PZ, TL, and ZL: data acquisition. PZ, DL, and FG: technical guidance. YZ and FG: data analysis and interpretation. FG: manuscript review—editing and supervision. All authors contributed to the article and approved the submitted version.

## Funding

This study was supported by grants from the National Natural Science Foundation of China (Nos. 81871393, 81971656, 62075156, and 61575140).

## Conflict of interest

The authors declare that the research was conducted in the absence of any commercial or financial relationships that could be construed as a potential conflict of interest.

## Publisher's note

All claims expressed in this article are solely those of the authors and do not necessarily represent those of their affiliated organizations, or those of the publisher, the editors and the reviewers. Any product that may be evaluated in this article, or claim that may be made by its manufacturer, is not guaranteed or endorsed by the publisher.
